# 24-Hour Movement Behaviors in Children with Chronic Disease and Their Healthy Peers: A Case-Control Study

**DOI:** 10.3390/ijerph19052912

**Published:** 2022-03-02

**Authors:** Rabha A. Elmesmari, John J. Reilly, James Y. Paton

**Affiliations:** 1 School of Medicine, College of Medical, Veterinary and Life Sciences, University of Glasgow, Glasgow G12 8QQ, UK; rabha.sulyman@glasgow.ac.uk; 2 Physical Activity for Health Group, University of Strathclyde, Glasgow G1 1QE, UK; john.j.reilly@strath.ac.uk

**Keywords:** children, type 1 diabetes mellitus, juvenile idiopathic arthritis, congenital heart disease, cystic fibrosis and 24 h movement, physical activity, objective measurement and non-movement behaviors

## Abstract

Background: Time spent in 24-h movement behaviors is important to health and wellbeing in childhood, but levels of these behaviors in children with chronic disease are unknown. Methods: A case-control-study included 80 children with chronic disease; 20 with type 1 diabetes mellitus (T1DM), 20 with juvenile idiopathic arthritis (JIA), 20 with congenital heart disease (CHD), 20 with cystic fibrosis (CF); pair-matched individually for age, sex, and timing of measures with 80 healthy children. Habitual time spent in movement behaviors and step counts were all measured with an activPAL accelerometer over 7 days. Comparisons against recommendations and differences between the groups were made. Results: Time spent in physical activity and step counts/day were significantly lower in T1DM and CHD groups compared to controls. Only 20/80 children with chronic disease and 29/80 controls met step count recommendations. Sedentary time was significantly higher in children with CF compared to controls. Time spent asleep was slightly greater in children with chronic disease, significant only for the JIA group. Sleep disruption was consistently greater in those with chronic disease, reaching significance for T1DM, CHD, and CF groups. Conclusions: For some groups of children with chronic disease, 24-h movement behaviors may differ substantially from recommendations, and slightly but systematically from their healthy peers. Optimizing levels of 24-h movement behaviors should confer a number of benefits for child health, development, and wellbeing.

## 1. Introduction

There is growing concern about low levels of physical activity (PA) in children and adolescents. Physical activity is defined as any “bodily movements” produced by skeletal muscles [1]; PA should not be confused with “exercise” [2], a subcategory of PA defined as planned, structured, repetitive bodily movement that aims to improve or maintain one or more components of physical fitness, such as swimming, or yoga (1–5). Most previous research on childhood PA has focused on healthy individuals. Our recent systematic review of objectively measured moderate-to-vigorous intensity PA (MVPA) in children with the chronic disease found only a relatively small amount of evidence (25 eligible studies across all groups with chronic disease and across all of childhood and adolescence), which suggested that habitual MVPA was consistently well below the recommended 60 min per day, though not very much lower than in healthy comparison groups. One exception was children being treated for malignancies where levels of MVPA were much lower [3]. A second systematic review of objectively measured MVPA in children or adolescents with obesity [4] also found clear evidence that obesity is associated with much lower than recommended MVPA, though only again slightly lower than in children from healthy comparison groups.

In recent years a paradigm shift has been taking place in the field of physical activity, with increasing emphasis on time spent in “24-h movement behaviors” (physical activity, sedentary behavior including sitting and screen time, sleep) arising from the evidence that time spent in the 24-h movement behaviors influences health, development, and well-being [5,6]. Evidence-based guidance for 24-h movement behaviors in childhood and adolescence was published in 2016 and 2017 [5,6,7], and WHO evidence-based guidance for the under 5 years of age was published by WHO in 2019 [8]. However, the concept of 24-h movement behaviors is relatively new, and quantifying the various movement behaviors with confidence is technically challenging with methods for analyzing “24-h data” still emerging [9,10].

Whether there are systematic differences in levels of 24-h movement behaviors in children with chronic disease and their healthy peers is unclear, but there are a number of medical, physiological, or psycho-social reasons why the behaviors may be affected by chronic disease. These include reasons related to the disease such as cardio-respiratory limitation or pain or factors related to anxiety arising either in the child or their parents about the effects of exercise on the disease.

The present study aimed to quantify levels of 24-h movement behaviors objectively in children with chronic disease and to test whether or not these differed from their healthy peers. The focus was on chronic diseases that were common and might plausibly have marked effects on the movement behaviors: Congenital heart disease (CHD) and cystic fibrosis (CF) are illnesses where cardio-respiratory limitations might limit PA; in juvenile inflammatory arthritis (JIA), inflamed joints may limit movement and cause pain and interference with both daily activities and sleep; in Type 1 diabetes (TIDM) limitation of PA might arise parentally imposed limitations to physical activity due to safety concerns while nocturnal wakening related to metabolic disturbance may interfere with sleep.

## 2. Materials and Methods

### 2.1. Participant Recruitment, Study Design, and Ethics

Patients were recruited from outpatient clinics at the Royal Hospital for Children, Glasgow, UK between August 2016 and November 2017. Our healthy control group was recruited from local schools and nurseries. The study had a paired design, with each patient matched with a healthy child (matched for age, gender, and week of measurement as these are the main influences on objectively measured physical activity and sedentary behavior in UK children) [11,12,13]. In all cases, the children with chronic diseases were recruited and measured while their diseases were relatively stable, and they were relatively well (attending school or nursery normally, attending hospital as out-patients and free from acute intercurrent illness or any acute exacerbation of their chronic disease). Full details for inclusion/exclusion criteria can be found here [14].

The study was approved by West of Scotland Research Ethics Committee 1 and NHS Greater Glasgow and Clyde Health Board. Parents of all participants provided informed written consent, and their children provided their informed assent—if applicable based on their age—to participate in the study prior to the collection of any study data.

Sex, age, disease duration, and a number of clinical variables related to each disease were recorded at the start of the study. Height and weight were measured to 0.1 cm and 0.1 kg, respectively, and Body Mass Index (BMI) was then calculated from the height and weight measures (kg/m^2^) and expressed relative to age and sex using the UK 1990 BMI reference data [15,16].

### 2.2. Objective Measurement of the 24-h Movement Behaviors

Furthermore, 24-hour movement behaviors were measured objectively using an activPAL^TM^ micro monitor (PAL Technologies Ltd., Glasgow, UK). The activPAL^TM^ micro is a small and lightweight (53 × 35 × 7 mm, 15.0 g) single unit tri-axial accelerometer/inclinometer that has been shown to be a valid and reliable tool for objective measurement of posture and motion during everyday activities in children [17,18,19]. The activPAL^TM^ uses algorithms to record time-spent sitting/lying, standing, walking, and transitions between these states. Children were asked to wear the monitor continuously, 24 h a day, for seven consecutive days. In an effort to reflect habitual levels of the behaviors, only children with ≥4 days with at least 10 h of waking wear time in a 24 h period including one weekend day and night, were included in the analysis.

During analysis of the 24-h data obtained from our participants, we first identified the nocturnal sleep period time (the time from sleep onset to sleep offset, including all sleep epochs and any wakefulness after sleep onset) [20], before considering any residual non-wear time, and thus waking wear monitoring time. For each participant, sleep duration for each day was determined by manual inspection of the event file and was identified as the time between the last transition from standing to sitting/lying at night to the subsequent first transition from sitting/lying to standing in the morning [20]. Non-wear time was identified from the parent activity log sheet, and located in activPAL^TM^ file. Once the non-wear time was identified, the missing data were calculated for each child and then excluded from the recording before any data analysis was made.

### 2.3. Comparisons against Recommended Levels of the 24-h Movement Behaviors

The activPAL can make objective measures of the amount of time spent in physical activity, number of steps per day (as a proxy for physical activity), time spent in sleep, time spent sedentary (sitting/lying down), and time spent standing but not moving. Of these behaviors, there are currently pediatric recommendations only for the number of steps per day and time spent in sleep. Time spent asleep and the number of steps per day were therefore compared against the appropriate age-specific recommendations: for steps per day the recommendation is 10,000–14,000 steps/day in children aged 4–6 years; 13,000–15,000 steps per day in boys and 11,000–12,000 steps per day in girls aged 6–11 years old to achieve an average of 60 min per day of MVPA [21]. For sleep duration, 10–13 h per night in children aged 3–4 years, and 9–11 h per night in children aged 5–13 years, with consistent bed and wake-up times are recommended [5,6].

### 2.4. Statistical Analysis and Study Power

All study variables were screened for normality: normally distributed data were summarized as mean (SD) while the median (interquartile range) was used for data not normally distributed. Our primary question in the statistical analyses was whether time spent in each of the movement behaviors, sleep disruption, and step count differed significantly between the children with each chronic disease and the controls. Because of the diversity of the nature of the four chronic diseases, each of the four disease groups was considered separately. Since children with chronic disease were matched pairwise with healthy controls (for age, sex, and time of year), and differences between patients and controls were normally distributed, paired t-tests were used to examine the significance of any differences between children with illness vs. their healthy controls.

At the time our study started, there were no data to guide sample size calculations of 24-h movement behaviors in children with chronic disease. Our systematic review [3] found that previous studies that examined differences in MVPA and sedentary time between children with chronic disease and healthy controls, found statistically significant differences with relatively small samples when using paired study designs like the present study: n between 7 pairs (7 patients and 7 healthy children) [22] and 38 pairs (38 children with chronic disease and 38 healthy children [23]. A number of studies of MVPA with paired designs [11,12,24,25] found that under 20 pairs were often sufficient to demonstrate significant differences between patients and controls, and so we aimed to recruit 20 pairs of participants in each of the chronic disease and control groups, providing four sets each of 20 paired comparisons.

In this study, the analysis was conducted using the IBM SPSS statistical software version 21 (IBM Corp., Armonk, NY, USA) and Microsoft Office Excel 2010 for Mac (Chicago, IL, US). Graphs were prepared using Graph Pad Prism version 6.00 software (San Diego, CA, USA).

## 3. Results

### 3.1. Characteristics of Participants

Of 136 potentially eligible children identified from outpatient clinics with chronic disease, a total of 99 (73%) agreed to take part. Of 111 healthy children identified as potentially healthy control children based on their age and sex, 89 (80%) agreed to take part.

Descriptive data including age, sex, weight, BMI z-score, and disease duration are listed in Table 1. There were no significant differences between any of the patient-control groups for age or BMI z-score.

### 3.2. Objectively Measured 24-h Movement Data: Levels of Each Behavior

Of the 91 eligible and recruited children with chronic disease, 80 (88%) provided sufficient activPAL data for inclusion (minimum of four 24-h periods with at least three valid nights including one at the weekend as noted above); similarly, 89 healthy control children 80 (90%) provided sufficient activPAL data for inclusion. The mean waking wear time was 13.2 h (SD 0.8) in the 80 children with chronic disease and 13.6 h (SD 0.6) in the 80 children recruited as healthy controls. In this study, the percentage of the total non-wear time per 24 h period was very low with a mean (SD) of 1.1% (SD 1) in the group with chronic disease and mean (SD) 0.6% (SD 0.5) in the healthy control group. More details are provided in Supplementary Table S1.

Table 2 shows the summary 24-h movement data (sitting/lying, standing, PA and sleep) and the percentage of these behaviors in a 24-h period for each of the groups with chronic disease and their healthy control groups. The mean number of sitting bouts per waking hours and the mean number of steps in 24-h period for each of the groups with chronic disease and their healthy control groups are also provided in Table 2.

Objectively measured 24-h movement data: comparisons between each group with chronic disease and healthy controls.

Table 2 shows comparisons between patient and control groups for each of the 24-h movement behaviors. Time spent sedentary was generally higher in patient than control groups. This reached significance only in the patients with CF, although was close to significance in those with CHD. The number of sitting bouts in waking hours was lower in the patient than control groups, reaching significance only in the group with JIA while being close to significance in those with T1DM. Time spent in standing was lower in the patient than the control groups but reached significance only in the groups with JIA and CF. Time spent in physical activity was significantly lower in the patient groups relative to healthy controls only in the groups with T1DM and CHD. The number of steps per day was lower in the patient than control groups for those with T1DM, JIA, and CHD, but this reached significance only in those with CHD. Sleep duration was similar between patient and matched control groups, except in those with JIA where sleep duration was slightly but significantly higher than in the control group. Sleep disruption (as measured by duration of wake episodes and movement during sleep time (minutes per night)) was significantly greater in the patient groups compared to controls for those with T1DM, CHD, and CF.

### 3.3. Objectively Measured 24-h Movement Data: Comparisons with Recommendations

As noted above, comparisons with recommendations were possible for the number of steps per day and for sleep duration. The mean number of steps per day was substantially less than the recommended value in all four groups with chronic disease and in their respective control groups. Only a total of 49/160 participants (n = 20 in children with chronic disease and 29 in healthy control groups) met current step count recommendations (Table 3). Mean duration of sleep was within recommendations for all four groups with chronic disease and for their four healthy control groups; for the individual-level data, a total of 23 participants (n = 10 in children with chronic disease and 13 in healthy control groups) had mean sleep duration below the sleep recommendations; from each group, the numbers not meeting the sleep recommendations are shown in Table 3.

### 3.4. Objectively Measured 24-h Movement Data: Comparisons with Recommendations

As noted above, comparisons with recommendations were possible for the number of steps per day and for sleep duration. The mean number of steps per day was substantially less than the recommended value in all four groups with chronic disease and in their respective control groups. Only a total of 49/160 participants (n = 20 in children with chronic disease and 29 in healthy control groups) met current step count recommendations (Table 3). Mean duration of sleep was within recommendations for all four groups with chronic disease and for their four healthy control groups; for the individual-level data, a total of 23 participants (n = 10 in children with chronic disease and 13 in healthy control groups) had mean sleep duration below the sleep recommendations; from each group, the numbers not meeting the sleep recommendations are shown in Table 3.

## 4. Discussion

Recent research in physical activity has increasingly emphasized time spent in “24-h movement behaviors” (physical activity, sedentary behavior including sitting and screen time, and sleep) as opposed to focusing on single components such as MVPA. This has arisen because of evidence that time spent in each of the 24-h movement behaviors can influence health, and the combination of behaviors can influence health. Despite intense recent interest in the new paradigm of “24-h movement behaviors” in children, there are currently few published data on time spent in these behaviors in children with chronic disease. In fact, having a chronic disease is often one of the exclusion criteria in studies.

Our present study reports 24 h movement behaviors in children with chronic diseases. We found that children with chronic disease were consistently slightly less physically active and slightly more sedentary than their healthy controls, and sedentary time was consistently more prolonged/less fragmented in the children with chronic diseases.

While the children recruited to patient groups in the present study were relatively well and living as outpatients, they and their families were still living with a substantial burden imposed by their chronic disease. For example, 40% (n = 8) of the children with T1DM were considered to have poor diabetes control at the time of recruitment; 35% were on insulin pumps while the others were on insulin pens or multiple insulin injections per day. For children with T1DM, the average duration from diagnosis to the time of enrolment in the study was 2.1 years. A total of four children with JIA were in remission and not receiving treatment at the time of enrolment in our study, but all other children with JIA were receiving injectable biological treatments with a mean of 3.2 years disease duration from diagnosis to the time of study enrolment. All children in the group with CHD had received substantial cardiac surgery, with a mean time from the last surgical procedure at the time of study enrolment of 3.5 years (SD = 2.2) years; 55% (n = 11) children were classified as having moderate-severe CHD; and 40% (n = 8) of children with CHD were still on medical treatments at the time of study enrolment. All children with CF were on treatment with the mean disease duration at the time of enrolment of 4.1 years (SD = 2.1) years. The lack of large differences in physical activity and the other 24-h movement behaviors between the children with chronic disease and the control groups in the present study is perhaps more of a reflection of the normalization of low levels of physical activity and high levels of sedentary time among modern children, rather than a reflection of markedly impaired physical activity in the groups with chronic disease. Sleep duration was generally within recommended values in all groups, but sleep in the children with chronic disease was slightly more disrupted than in the healthy children. Differences between children with chronic disease and their healthy peers may be different as disease severity increases, and may also increase with age. For example, longitudinal studies in healthy children and adolescents have shown that from around age 6–7 years (the mean age of participants in the present study), time spent sedentary increases and this displaces physical activity throughout childhood and adolescence, and by adolescence, it also displaces sleep [11,12].

The main strength of the present study was the novelty of the research questions and the populations of children studied in the context of a paucity of objectively measured and fully quantitative 24-h movement data from children in general and an even more severe lack of data from children with chronic disease. Further strengths were our ability to make objective measures of a number of the 24-h movement behaviors (time spent sitting, standing, moving, and sleeping), to control for the key known determinants of these behaviors at this age in the UK (age, gender, season) and to make pair-wise comparisons of the behaviors with closely matched healthy controls. We were also able to allow for the fact that children spend a good deal of time standing but not moving. To date, time spent standing has generally not been considered in earlier studies, and it has possibly been misclassified as other forms of movement behavior.

New techniques for analyzing 24-h movement behavior data are emerging [27]. We did not use them in the present study in part because they are still in development. We also wanted to focus on levels of individual behaviors in children with chronic diseases and healthy children for those behaviors where recommendations have been published.

One potential weakness of the present study is a possible lack of generalizability to other chronic diseases, or to children with the same chronic diseases but different levels of severity (either more or less severe) then recruited to the present study, particularly children with even more severe/advanced forms (or during disease exacerbations).

Our systematic reviews [3,4] suggested that paired comparisons with 20 pairs can provide sufficient power. Some of the differences between groups that were actually observed were relatively small and larger studies may be required to determine the statistical significance of differences between some of the groups. Our analyses across the whole study involved 30 paired comparisons between children with chronic disease vs. healthy controls (six behaviors compared between the four chronic disease groups vs. controls, and all children with chronic disease vs. all healthy controls). Using *p* =< 0.05 and with 30 paired comparison tests then 1–2 (1.5) significant between-group differences would be normally expected by chance. We actually observed 13 statistically significant paired differences, substantially more than would have been expected by chance alone. Correction for multiple testing is not straightforward, and the most widely used method in the literature, the Bonferroni Correction, has been criticized as being overly conservative, overused, and of limited value where comparisons are not independent, as in the present study [28]. We, therefore, did not use any formal methods of correction for multiple testing, but the limitations of multiple testing are acknowledged. However, we would argue that the significance of the main findings of the present study does not depend on the statistical significance of between-group comparisons alone. In our view, the clinical significance of the findings goes beyond the statistical significance of between-group comparisons. Specifically, all four chronic disease groups were characterized by low levels of physical activity, high levels of sedentary behavior but apparently adequate levels of sleep. This should be contrasted with disease-specific recommendations for all of the chronic disease groups that suggest levels of, for example, PA should be as high for children with chronic disease as for healthy children [29,30,31,32,33].

A further weakness was our inability to measure screen time–a subset of sedentary behavior. Identical screen-time recommendations for children have been made for both healthy children and those with chronic disease, but there are currently no objective methods available for measuring screen time exposure in children and the accuracy of subjective (parent-report) methods is unknown. If this methodological limitation could be overcome, then a more comprehensive picture of 24-h movement behaviors would become possible. There are also some other technical limitations to consider. For example, we were unable to measure MVPA in the present study, as there are currently no algorithms for converting activPAL-measured movement to MVPA in school-age children.

## 5. Conclusions

In conclusion, the present study suggests that 24-h movement behaviors in children with chronic disease give cause for concern, and are generally slightly worse than in their healthy peers. Optimizing levels of the 24-h movement behaviors should confer a number of benefits for child health, development, and wellbeing [5,6] and so improving 24-h movement behaviors should be considered as part of the management of common childhood chronic diseases in the future.

## Figures and Tables

**Table 1:** Characteristics of children with chronic disease and healthy controls age- and sex-matched groups. Data are presented as mean (SD).

Characteristics	T1DM (n = 20)	HC (n = 20)	JIA (n = 20)	HC (n = 20)	CHD (n = 20)	HC (n = 20)	CF (n = 20)	HC (n = 20)	CD (n = 80)	HC (n = 80)
Age years	7.4 (1.9)	7.3 (1.8)	6.7 (2.1)	6.7 (2.1)	6.0 (2.2)	6.1 (2.1)	6.7 (2.0)	6.9 (1.9)	6.9 (2.0)	6.8 (1.9)
Sex F:M	9:11	9:11	12:8	12:8	10:10	10:10	12:8	12:8	43:37	43:37
Weight kg	25.6 (6.3)	28.7 (8.3)	23.4 (6.0)	25.7 (6.4)	24.0 (8.5)	25.1 (5.3)	24.8 (6.5)	22.6 (6.2)	24.4 (6.8)	25.5 (6.5)
Weight-Z-score	0.1 (1.0)	0.8 (1.4)	0.2 (1.2)	1.1 (1.2)	0.6 (1.0)	1.4 (0.9)	0.4 (2.1)	−0.1 (0.7)	0.3 (1.3)	0.8 (0.7)
Height cm	122.6 (12.8)	125.4 (13.2)	119.6 (13.7)	123.2 (10.0)	117.1 (15.6)	118.7 (15.3)	119.7 (12.5)	117.0 (12.8)	119.7 (13.6)	121.1 (12.8)
Height-Z-score	−0.1 (1.1)	0.4 (1.8)	0.2 (1.9)	1.2 (1.0)	0.4 (1.1)	0.5 (1.0)	0.2 (2.0)	−0.4 (0.8)	0.7 (1.0)	0.5 (0.8)
BMI kg/m^2^	16.6 (2.2)	17.8 (2.5)	16.2 (1.9)	17.3 (2.5)	17.0 (2.2)	17.2 (1.2)	17.3 (3.9)	16.2 (1.6)	16.8 (2.6)	17.1 (2.0)
BMI Z-score ^1^	0.3 (1.2)	0.9 (1.0)	0.1 (1.1)	0.6 (1.4)	0.5 (1.0)	0.8 (1.1)	0.2 (2.7)	0.2 (0.8)	0.3 (1.5)	0.6 (0.8)
BMI category ^2^										
Normal weight n (%)	17 (85%)	16 (80%)	17 (85%)	14 (70%)	16 (80%)	17 (85%)	14 (70%)	19 (95%)	64 (80%)	66 (82%)
Overweight n (%)	2 (10%)	2 (10%)	1 (5%)	5 (25%)	3 (15%)	3 (15%)	3 (15%)	1 (5%)	9 (11%)	11 (14%)
Obese n (%)	1 (5%)	2 (10%)	2 (10%)	1 (5%)	1 (5%)	0 (0%)	3 (15%)	0 (0%)	7 (9%)	3 (4%)
Disease duration Years	2.12 (1.4)	-	3.2 (2.0)	-	3.5 (2.20)	-	6.5 (2.0)	-	3.8 (1.9)	-

BMI—body mass index; CD—chronic disease; CHD—congenital heart disease; CF—cystic fibrosis; JIA—juvenile idiopathic arthritis; T1DM—type1 diabetes mellitus. Data are presented as means and standard deviation SD unless otherwise specified. ^1^ Based on World Health Organization Growth Reference data from 2007 [26]. ^2^ Based on age- and gender-specific cut-offs defined body mass index at 18.5, 25, and 30 kg/m^2^ at age 18 years [15,16].

**Table 2:** 24-h movement behavior variables in children with chronic disease and their healthy control groups; data are presented as means and (SD).

Variables	T1DM (n = 20)	HC (n = 20)	JIA (n = 20)	HC (n = 20)	CHD (n = 20)	HC (n = 20)	CF (n = 20)	HC (n = 20)	CD (n = 80)	HC (n = 80)
Time spent sitting (h)	8.3 (1.7)	7.8 (0.8)	7.6 (1.1)	7.4 (1.0)	8.1 (1.7)	7.4 (0.9)	8.1 (1.0) *	7.2 (0.9) *	8.1 (1.4) *	7.4 (0.9) *
No. of sitting bouts (n)	210 (56)	243 (93)	236 (75) **	319 (87) **	241 (61)	298 (82)	249 (31)	261 (83)	245 (68) **	277 (88) **
Time spent standing (h)	3.2 (0.7)	3.5 (0.6)	3.2 (0.9) *	3.8 (0.8) *	3.2 (0.8)	3.6 (0.5)	2.9 (0.5) ***	3.7 (0.7) ***	3.1 (0.7) ***	3.7 (0.7) ***
Time spent in PA (h)	1.7 (0.7) *	2.2 (0.4) *	2.3 (0.9)	2.6 (0.3)	1.9 (0.9) *	2.5 (0.4) *	2.4 (0.7)	2.4 (0.4)	2.1 (0.8) **	2.5 (0.4) **
Steps per 24 h	8201 (3231)	9903 (1657)	10,428 (4139)	11,608 (1561)	8533 (4712) *	11,249 (2320) *	11,395 (4169)	11,050 (2128)	9485 (4148) *	10,959 (2024) *
Time spent in sleep (h)	10.2 (0.7)	9.7 (0.6)	10.2 (0.7)	9.7 (0.6)	10.2 (0.9)	9.7 (0.5)	9.7 (0.9)	9.9 (0.5)	10.1 (0.8) **	9.7 (0.6) **

CD—chronic disease; CHD—congenital heart disease; CF—cystic fibrosis; JIA—juvenile idiopathic arthritis; h—hour; T1DM—type1 diabetes mellitus. Combined data represented total children with chronic disease (CD n = 80, mean age 6.9 years (SD = 2.0), and F:M 43:37) as one group and total healthy controls n = 80, mean age 6.8 (SD = 1.9) and F:M 43:37) as one group. Data are presented as means and standard deviation SD unless otherwise specified. Paired *t* test compared between children with chronic disease and healthy children control age- and sex-matched; * *p* =< 0.05, ** *p* =< 0.005, and *** *p* =< 0.0005.

**Table 3:** 24-h movement behavior variables comparisons with recommendations in children with chronic disease and their healthy control groups; data are presented as numbers.

Variables	T1DM (n = 20)	HC (n = 20)	JIA (n = 20)	HC (n = 20)	CHD (n = 20)	HC (n = 20)	CF (n = 20)	HC (n = 20)	CD (n = 80)	HC (n = 80)
No. of participants not meeting steps recommendation	17	16	12	11	16	12	15	12	60	51
No. of participants not meeting sleep recommendation	3	4	0	5	3	2	4	2	10	13

CHD—congenital heart disease; CF—cystic fibrosis; JIA—juvenile idiopathic arthritis; T1DM—type1 diabetes mellitus. Combined data represented total children with chronic disease (CD n = 80, mean age 6.9 years (SD = 2.0), and F:M 43:37) as one group and total healthy controls n = 80, mean age 6.8 (SD = 1.9) and F:M 43:37) as one group. Steps recommendations per day 10,000–14,000 steps/day in children aged 4–6 years, 13,000–15,000 steps per day in boys and 11,000–12,000 steps per day in girls aged 6–11 years old to achieve an average of 60 min per day of MVPA [21]. Sleep duration recommendations 10–13 h per night in children aged 3–4 years, and 9–11 h per night in children aged 5–13 years, with consistent bed and wake-up times [5,6]. Data are presented as numbers.

## Data Availability

An anonymized dataset will be available from the corresponding author on request.

## Supplementary Materials

The following are available online at https://www.mdpi.com/article/10.3390/ijerph19052912/s1, Supplementary Table S1: Accelerometer results based on 24-h data.

## References

[1] Caspersen C.J., Powell K.E., Christenson G.M. (1985). Physical activity, exercise, and physical fitness: Definitions and distinctions for health-related research. Public Health Rep..

[2] Bailey R.C., Olson J., Pepper S.L., Porszasz J., Barstow T.J., Cooper D.M. (1995). The level and tempo of children’s physical activities: An observational study. Med. Sci. Sports Exerc..

[3] Elmesmari R., Reilly J.J., Martin A., Paton J.Y. (2017). Accelerometer measured levels of moderate-to-vigorous intensity physical activity and sedentary time in children and adolescents with chronic disease: A systematic review and meta-analysis. PLoS ONE.

[4] Elmesmari R., Martin A., Reilly J.J., Paton J.Y. (2018). Comparison of accelerometer measured levels of physical activity and sedentary time between obese and non-obese children and adolescents: A systematic review. BMC Pediatr..

[5] Tremblay M.S., Carson V., Chaput J.P., Connor Gorber S., Dinh T., Duggan M., Faulkner G., Gray C.E., Gruber R., Janson K. (2016). Canadian 24-Hour Movement Guidelines for Children and Youth: An Integration of Physical Activity, Sedentary Behaviour, and Sleep. Appl. Physiol. Nutr. Metab..

[6] Tremblay M.S., Chaput J.P., Adamo K.B., Aubert S., Barnes J.D., Choquette L., Duggan M., Faulkner G., Goldfield G.S., Gray C.E. (2017). Canadian 24-Hour Movement Guidelines for the Early Years (0–4 years): An Integration of Physical Activity, Sedentary Behaviour, and Sleep. BMC Public Health.

[7] Okely D.A., Tremblay S.M., Reilly J.J., Draper E.C., Bull F. (2018). Physical activity, sedentary behaviour, and sleep: Movement behaviours in early life. Lancet Child Adolesc. Health.

[8] World Health Organization (2019). Guidelines on Physical Activity, Sedentary Behaviour and Sleep for Children under 5 Years of Age.

[9] Cliff D.P., McNeill J., Vella S.A., Howard S.J., Santos R., Batterham M., Melhuish E., Okely A.D., de Rosnay M. (2017). Adherence to 24-Hour Movement Guidelines for the Early Years and associations with social-cognitive development among Australian preschool children. BMC Public Health.

[10] Walsh J.J., Barnes D.J., Cameron D.J., Goldfield G.S., Chaput J.P., Tremblay M.S. (2018). Associations between 24 hour movement behaviours and global cognition in US children: A cross-sectional observational study. Lancet Child Adolesc. Health.

[11] Farooq M.A., Parkinson K.N., Adamson A.J., Pearce M.S., Reilly J.K., Hughes A.R., Janssen X., Basterfield L., Reilly J.J. (2018). Timing of the decline in physical activity in childhood and adolescence: Gateshead Millennium Cohort Study. Br. J. Sports Med..

[12] Janssen X., Mann K.D., Basterfield L., Parkinson K.N., Pearce M.S., Reilly J.K., Adamson A.J., Reilly J.J. (2016). Development of sedentary behavior across childhood and adolescence: Longitudinal analysis of the Gateshead Millennium Study. Int. J. Behav. Nutr. Phys. Act..

[13] Kwon S., Lee J., Carnethon M.R. (2015). Developmental trajectories of physical activity and television viewing during adolescence among girls: National Growth and Health Cohort Study. BMC Public Health.

[14] Sulyman R. (2019). Integrated Movement Behaviours in Children with Chronic Disease: An Observational Case-Control Study. Master’s Thesis.

[15] Cole T.J., Bellizzi M.C., Flegal K.M., Dietz W.H. (2000). Establishing a standard definition for child overweight and obesity worldwide: International survey. BMJ.

[16] Cole T.J., Flegal K.M., Nicholls D., Jackson A.A. (2007). Body mass index cut offs to define thinness in children and adolescents: International survey. BMJ.

[17] Davies G., Reilly J.J., McGowan A.J., Dall P.M., Granat M.H., Paton J.Y. (2012). Validity, practical utility, and reliability of the activPAL in preschool children. Med. Sci. Sports Exerc..

[18] Aminian S., Hinckson E.A. (2012). Examining the validity of the ActivPAL monitor in measuring posture and ambulatory movement in children. Int. J. Behav. Nutr. Phys. Act..

[19] Van Loo C.M.T., Okely A.D., Batterham M., Hinkley T., Ekelund U., Brage S., Reilly J.J., Peoples G.E., Jones R., Janssen X. (2016). Predictive Validity of a Thigh-Worn Accelerometer METs Algorithm in 5-to 12-Year-old Children. J. Phys. Act. Health.

[20] Alghaeed Z., Reilly J.J., Chastin S.F., Martin A., Davies G., Paton J.Y. (2013). The influence of minimum sitting period of the ActivPAL on the measurement of breaks in sitting in young children. PLoS ONE.

[21] Tudor-Locke C., Craig C.L., Beets M.W., Belton S., Cardon G.M., Duncan S., Hatano Y., Lubans D.R., Olds T.S., Raustorp A. (2011). How many steps/day are enough? for children and adolescents. Int. J. Behav. Nutr. Phys. Act..

[22] Aznar S., Webster A.L., San Juan A.F., Chamorro-Vina C., Mate-Munoz J.L., Moral S., Perez M., Garcia-Castro J., Ramirez M., Madero L. (2006). Physical activity during treatment in children with leukemia: A pilot study. Appl. Physiol. Nutr. Metab. Physiol. Appl. Nutr. Metab..

[23] Tan S.Y., Poh B.K., Chong H.X., Ismail M.N., Rahman J., Zarina A.L., Juraida A.R., Tahir A., Ruzita A.T., Roslee R. (2013). Physical activity of pediatric patients with acute leukemia undergoing induction or consolidation chemotherapy. Leuk. Res..

[24] Cooper A.R., Goodman A., Page A.S., Sherar L.B., Esliger D.W., van Sluijs E.M., Andersen L.B., Anderssen S., Cardon G., Davey R. (2015). Objectively measured physical activity and sedentary time in youth: The International children’s accelerometry database (ICAD). Int. J. Behav. Nutr. Phys. Act..

[25] Basterfield L., Adamson A.J., Frary J.K., Parkinson K.N., Pearce M.S., Reilly J.J., Gateshead Millennium Study Core Team (2011). Longitudinal study of physical activity and sedentary behavior in children. Pediatrics.

[26] De Onis M., Onyango A.W., Borghi E., Siyam A., Nishida C., Siekmann J. (2007). Development of a WHO growth reference for school-aged children and adolescents. Bull. World Health Organ..

[27] Chastin S.F., Palarea-Albaladejo J., Dontje M.L., Skelton D.A. (2015). Combined effects of time spent in physical activity, sedentary behavior and sleep on adiposity and cardiometabolic health markers: A novel compositional data analysis approach. PLoS ONE.

[28] Green J., Nicky B. (1998). Qualitative research and evidence based medicine. BMJ.

[29] Fredriksen P.M., Kahrs N., Blaasvaer S., Sigurdsen E., Gundersen O., Roeksund O., Norgaand G., Vik J.T., Soerbye O., Ingjer E. (2000). Effect of physical training in children and adolescents with congenital heart disease. Cardiol. Young.

[30] NICE Guideline (2015). Diabetes (Type 1 and Type 2) in Children and Young People: Diagnosis and Management. NICE Guideline [Online]. https://www.nice.org.uk/guidance/ng18.

[31] Pate R.R., Pratt M., Blair S.N., Haskell W.L., Macera C.A., Bouchard C., Buchner D., Ettinger W., Heath G.W., King A.C. (1995). Physical activity and public health. A recommendation from the Centers for Disease Control and Prevention and the American College of Sports Medicine. JAMA.

[32] Takken T., Giardini A., Reybrouck T., Gewillig M., Hovels-Gurich H.H., Longmuir P.E., McCrindle B.W., Paridon S.M., Hager A. (2012). Recommendations for physical activity, recreation sport, and exercise training in paediatric patients with congenital heart disease: A report from the Exercise, Basic & Translational Research Section of the European Association of Cardiovascular Prevention and Rehabilitation, the European Congenital Heart and Lung Exercise Group, and the Association for European Paediatric Cardiology. Eur. J. Prev. Cardiol..

[33] Takken T., van der Net J., Kuis W., Helders P.J. (2003). Physical activity and health related physical fitness in children with juvenile idiopathic arthritis. Ann. Rheum. Dis..

