# Supplementary material for: 24-Hour Movement Behaviors in Children with Chronic Disease and Their Healthy Peers: A Case-Control Study
Source: Int J Environ Res Public Health. 2022 Mar 2;19(5):2912. doi: 10.3390/ijerph19052912 (PMC8910254; doi:10.3390/ijerph19052912)
Supplement: Supplementary file 1 [file ijerph-19-02912-s001.zip › ijerph-1541175-supplementary.pdf]

Supplementary Table S1: Accelerometer results based on 24-h data. Data are presented as means and SD

Variables	T1DM (n =20)	HC (n =20)	JIA (n =20)	HC (n =20)	CHD (n =20)	HC (n =20)	CF (n =20)	HC (n =20)
**No. of monitoring days**	7.4 (0.9)	6.8 (0.9)	6.8 (1.0)	7.1 (0.7)	7.4 (0.8)	7.0 (0.9)	6.6 (1.4)	6.9 (0.6)
**No. of monitoring nights**	6.9 (0.4)	6.6 (0.7)	6.7 (0.7)	7.0 (0.6)	7.1 (0.9)	6.0 (0.9)	6.5 (1.5)	7.1 (0.5)
**Monitoring time during waking hours/day**	13.2 (0.7)	13.6 (0.6)	13.0 (0.7)	13.9 (0.7)	13.2 (1.0)	13.5 (0.6)	13.5 (0.7)	13.4 (0.4)
**Non-wear time during waking time minutes/day**	20.4 (13.9)	18.1 (10.2)	15.8 (14.8)	12.3 (10.8)	17.7 (20.4)	7.4 (5.9)	9.8 (8.7)	9.9 (8.1)
**Non-wear time during sleep time minutes/day**	0	0	0	0	0	0	0	0
**% of total non-wear time per 24 hours period**	1.3 (1.0)	0.3 (0.6)	1.1 (1.0)	0.9 (0.7)	1.2 (1.4)	0.5 (0.4)	0.7 (0.6)	0.6 (0.6)

BMI – body mass index; CHD – congenital heart disease; CF – cystic fibrosis; JIA – juvenile idiopathic arthritis; T1DM – type1 diabetes mellitus.

Data are presented as means and standard deviation SD unless otherwise specified.
